# Population‐based cohort study of the impact on postoperative mortality of anastomotic leakage after anterior resection for rectal cancer

**DOI:** 10.1002/bjs5.50106

**Published:** 2018-10-15

**Authors:** P. Boström, M. M. Haapamäki, J. Rutegård, P. Matthiessen, M. Rutegård

**Affiliations:** ^1^ Surgery Unit, Department of Surgical and Perioperative Sciences Umeå University Umeå Sweden; ^2^ Department of Surgery, Faculty of Medicine and Health, School of Health and Medical Sciences Örebro University Örebro Sweden

## Abstract

**Background:**

Anastomotic leakage following anterior resection for rectal cancer may result in death. The aim of this study was to yield an updated, population‐based estimate of postoperative mortality and evaluate possible interacting factors.

**Methods:**

This was a retrospective national cohort study of patients who underwent anterior resection between 2007 and 2016. Data were retrieved from a prospectively developed database. Anastomotic leakage constituted exposure, whereas outcome was defined as death within 90 days of surgery. Logistic regression analyses, using directed acyclic graphs to evaluate possible confounders, were performed, including interaction analyses.

**Results:**

Of 6948 patients, 693 (10·0 per cent) experienced anastomotic leakage and 294 (4·2 per cent) underwent reintervention due to leakage. The mortality rate was 1·5 per cent in patients without leakage and 3·9 per cent in those with leakage. In multivariable analysis, leakage was associated with increased mortality only when a reintervention was performed (odds ratio (OR) 5·57, 95 per cent c.i. 3·29 to 9·44). Leaks not necessitating reintervention did not result in increased mortality (OR 0·70, 0·25 to 1·96). There was evidence of interaction between leakage and age on a multiplicative scale (*P* = 0·007), leading to a substantial mortality increase in elderly patients with leakage.

**Conclusion:**

Anastomotic leakage, in particular severe leakage, led to a significant increase in 90‐day mortality, with a more pronounced risk of death in the elderly.

## Introduction

Every anastomosis has a risk of anastomotic leakage. For reasons only partly understood, this risk is greater for extraperitoneal colorectal anastomoses than for other enteric anastomoses^1^. Anastomotic leakage can lead to faecal peritonitis, sepsis and multiple organ failure, and is thus associated with substantial morbidity and mortality. Modern population‐based prospective studies^2^ ^3^ on anterior resection for rectal cancer have estimated the risk of anastomotic leakage, depending on whether or not a stoma was created, to be 7·8–9 and 11·6–12 per cent respectively. The mortality rate for patients with leakage was 5·7–7 per cent in these studies.

Following the introduction of total mesorectal excision for rectal cancer, the incidence of anastomotic leakage increased initially^4^ ^5^. More recently, however, both the incidence and severity of anastomotic leak have decreased owing to a greater understanding of the risk factors for anastomotic leakage, the impact of protective measures, especially the construction of a diverting stoma, which mitigates the effects of leakage^2^ ^5^, ^6^, ^7^, ^8^, ^9^, and improved postoperative management. Modern intensive care can support vital functions for longer than the traditional 30‐day period during which postoperative complications (including death) were considered to occur. For this reason, more recent studies^10^ ^11^ on postoperative complications have used an extended interval of 90 days so that the risk of adverse outcomes after surgery is not underestimated.

The aim of this study was to make an updated, population‐based estimate of the incidence of anastomotic leakage and associated postoperative mortality. Possible interacting factors were also evaluated, including the use of a diverting stoma.

## Methods

This was a retrospective study of patients with rectal cancer included in the Swedish Cancer Registry. Since 2007, the Regional Oncological Centre in each healthcare region in Sweden has provided data for the national Swedish Colorectal Cancer Registry. All patients with colorectal cancer in Sweden have been reported to this registry, which has frequently been checked against the National Cancer Registry to ensure completeness. Data on patient characteristics, surgery, postoperative treatment, pathological assessment and follow‐up for 5 years are recorded, with new cases stemming from clinicians and pathologists. The registry has been validated on a number of occasions, showing a level of completeness of 97 per cent regarding rectal cancer surgery^12^. The registry uses several categories for surgical complications, including wound infection, wound dehiscence, intra‐abdominal infection, postoperative bleeding, anastomotic leakage, stoma complication, urinary catheter at discharge, or not specified.

Patients were included in the present study if their operation was registered as anterior resection for rectal cancer and performed in 2007–2016. Rectal cancer was defined as histologically proven adenocarcinoma, with its lower border within 15 cm of the anal verge, measured by rigid sigmoidoscopy. No exclusion criteria were used.

### Exposure and outcome

Primary exposure was defined as anastomotic leakage within 30 days of surgery or intervention for anastomotic leak, as recorded in the registry. Anastomotic leak was defined as leakage of the colorectal anastomosis, pelvic abscess or rectovaginal fistula^13^. As secondary exposure, leakage categorized as leaks not necessitating reintervention, as well as those where reintervention was used, was examined, to outline groups of patients with different severity of anastomotic leakage. Reoperation and radiologically performed drainage were defined as reinterventions by the registry, without defining which reintervention was used.

Postoperative mortality was defined as a registered death in the Swedish total population registry, within 90 days of surgery. This dichotomous outcome variable was used for logistic regression analyses.

### Hypotheses

The main hypothesis was that anastomotic leakage is associated with an increased 90‐day mortality rate. A dose–response relationship was expected between the severity of anastomotic leakage and mortality, and that leakage would interact with patient‐related and operative risk factors. In addition, the aim was to calculate an updated, population‐based estimate of the relative risk of death within 90 days after anastomotic leakage, in a recent time period.

### Statistical analyses

Univariable associations were evaluated with the χ^2^ test and Fisher's exact test, as appropriate. Unconditional logistic regression was performed to yield single estimates, measured as odds ratios (ORs) with 95 per cent c.i. of the independent risk of postoperative death at 90 days.

To adjust for confounding the concept of directed acyclic graphs^14^, ^15^, ^16^ was used, and biologically mechanistic reasoning as to how different possible confounders influence exposure and outcome. This model (*Fig*. *S1*, supporting information) is considered an unbiased measure of the total effect of anastomotic leakage and reintervention for leakage on postoperative mortality. It could be calculated by adjusting for age (continuous), hospital volume (divided by tertiles), ASA grade (I, II, III–IV), presence of a diverting stoma (yes or no) and intraoperative blood loss (using the median: 300 ml or less *versus* more than 300 ml). Mixed models were used to accommodate for clustering within hospitals. The hypothetical dose–response relationship was evaluated by grading leakage severity according to need for reintervention. Multiplicative interaction analyses were conducted of overall anastomotic leakage with every co‐variable, using a backward‐elimination procedure. To visualize the interaction results, predicted probabilities of death were calculated and plotted in graphical form.

The regression models were analysed using the Hosmer–Lemeshow test for goodness‐of‐fit, and tested for collinearity by calculating the variance inflation factor and evaluating *P* values. A complete‐case analysis was used, thereby excluding observations with missing data. All tests for significance were two‐sided, with the level of significance set at 5 per cent. All analyses were conducted in STATA® 13.1 (StataCorp, College Station, Texas, USA).

## Results

Some 6948 patients were identified using the registry. The majority were men (4103, 59·1 per cent) with only mild systemic disease (ASA grade II). The median age was 67 years, and half the population was aged 60–74 years. Tumour stages I–III were roughly equally common, whereas a minority of tumours were stage IV cancers. Most tumours were located at 7–12 cm, and only 518 (7·5 per cent) were 6 cm or less from the anal verge. A total of 121 patients (1·7 per cent) died within 90 days of surgery (median 25 (i.q.r. 6–46) days).

A diverting stoma was constructed in 5331 patients (76·7 per cent). Some 1262 patients (18·2 per cent) were operated on laparoscopically. Of these operations, 247 were converted to open surgery (conversion rate 19·6 per cent). A J pouch or side‐to‐end anastomosis was favoured over end‐to‐end anastomosis. *Table* 1 outlines the frequencies of patient characteristics, operative data and postoperative complications, stratified by mortality.

**Table 1:** Clinical variables stratified by mortality within 90 days after surgery, for patients undergoing anterior resection for rectal cancer in Sweden 2007–2016

	Postoperative mortality within 90 days		
	No (*n* = 6827)	Yes (*n* = 121)	*P* ¶
Anastomotic leakage			< 0·001
No	6161 (90·2)	94 (77·7)	
Yes	666 (9·8)	27 (22·3)	
Reintervention for leakage			< 0·001
No	6555 (96·0)	99 (81·8)	
Yes	272 (4·0)	22 (18·2)	
Age (years)			< 0·001
< 65	2668 (39·1)	11 (9·1)	
65–75	2851 (41·8)	44 (36·4)	
> 75	1308 (19·2)	66 (54·5)	
ASA fitness grade			< 0·001
I	1659 (24·3)	6 (5·0)	
II	3914 (57·3)	66 (54·5)	
III–IV	1138 (16·7)	48 (39·7)	
Tumour stage (pTNM)			0·651
I	1822 (26·7)	30 (24·8)	
II	1886 (27·6)	37 (30·6)	
III	2335 (34·2)	41 (33·9)	
IV	468 (6·9)	5 (4·1)	
Radiotherapy			0·004
Short course*	1983 (29·0)	38 (31·4)	
Long course†	488 (7·2)	1 (0·8)	
Other	21 (0·3)	1 (0·8)	
None	4335 (63·5)	81 (66·9)	
Tumour level (cm)‡			0·336
≤ 6	509 (7·5)	9 (7·4)	
7–12	4512 (66·1)	73 (60·3)	
13–15	1806 (26·5)	39 (32·2)	
Type of anastomosis			0·173
End‐to‐end	1906 (27·9)	40 (33·1)	
Side‐to‐end/J pouch	4496 (65·9)	71 (58·7)	
Surgical technique			0·039
Open surgery	5545 (81·2)	99 (81·8)	
Laparoscopy, not converted	1003 (14·7)	12 (9·9)	
Laparoscopy, converted	238 (3·5)	9 (7·4)	
Diverting stoma			0·015
Yes	5249 (76·9)	82 (67·8)	
No	1578 (23·1)	39 (32·2)	
Blood loss (ml)			0·013#
≤ 300	3413 (50·0)	52 (43·0)	
> 300	3414 (50·0)	69 (57·0)	
Surgical complication§			< 0·001
No	5402 (79·1)	74 (61·2)	
Yes	1425 (20·9)	47 (38·8)	
Neurological complication			0·038
No	6811 (99·8)	119 (98·3)	
Yes	16 (0·2)	2 (1·7)	
Non‐surgical infection			< 0·001
No	6383 (93·5)	92 (76·0)	
Yes	444 (6·5)	29 (24·0)	
Cardiovascular complication			< 0·001
No	6653 (97·5)	87 (71·9)	
Yes	174 (2·5)	34 (28·1)	

Values in parentheses are percentages; percentages may not sum to 100 owing to missing values for some variables.

* 5 × 5 Gy;

† 1·8–2·0 Gy to a total of 46–50·4 Gy.

‡ Measured from anal verge.

§ Surgical complication noted in the registry as any of the following: wound infection, wound dehiscence, intra‐abdominal infection, postoperative bleeding, anastomotic leakage, stoma complication, urinary catheter at discharge or not specified.

¶ χ^2^ or Fisher's exact test, except

# Mann–Whitney *U* test (blood loss analysed as a continuous variable).

### Anastomotic leakage and postoperative mortality

In total, 693 of 6948 patients (10·0 per cent) developed anastomotic leakage. The mortality rate in these patients was 3·9 per cent, compared with 1·5 per cent in patients without leakage. In univariable logistic regression analysis, anastomotic leak was associated with increased mortality (OR 2·66, 95 per cent c.i. 1·72 to 4·11). This association was similar in multivariable analysis (OR 2·64, 1·65 to 4·22) (*Table* 2). In the 294 patients (4·2 per cent) who underwent reintervention for leakage, the mortality rate was 7·5 per cent.

**Table 2:** Frequency and logistic regression analysis of postoperative mortality within 90 days after anastomotic leak and reintervention for leakage

	No. of leaks	No. of deaths/leak*	Univariable analysis	Multivariable analysis‡
			Odds ratio†	Odds ratio†
All leaks	693	27 (3·9)	2·66 (1·72, 4·11)	2·64 (1·65, 4·22)
Leak without reintervention	399	5 (1·3)	0·83 (0·34, 2·06)	0·70 (0·25, 1·96)
Leak with reintervention	294	22 (7·5)	5·30 (3·28, 8·57)	5·57 (3·29, 9·44)

Values in parentheses are

* percentages and

† 95 per cent confidence intervals.

‡ Adjusted for age, ASA grade, diverting stoma, hospital volume, intraoperative blood loss and clustering within hospitals.

In the secondary analysis of leakage severity, only anastomotic leakage with reintervention was associated with increased mortality (OR 5·57, 95 per cent c.i. 3·29 to 9·44). For leakage without intervention, no statistically significant association was observed (OR 0·70, 0·25 to 1·96) (*Table* 2).

### Interaction analyses

**Figure 1:**
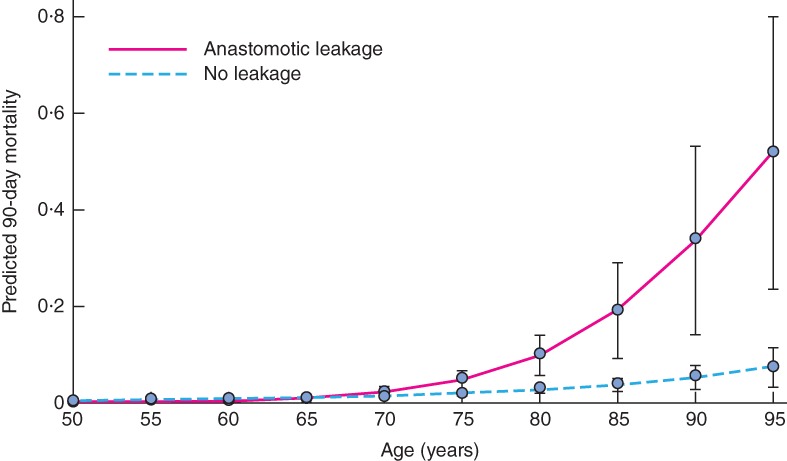
Predicted 90‐day postoperative mortality after anterior resection for rectal cancer according to age, for patients with and without anastomotic leakage

On a multiplicative scale, stepwise interaction analyses were performed to eliminate non‐significant variables (hospital volume, intraoperative bleeding, ASA grade and diverting stoma). The only interaction variable that remained was age (OR 1·09, 95 per cent c.i. 1·02 to 1·17). The variable age had a high variance inflation factor (41·5), but a low *P* value (*P* = 0·007), and was therefore retained in the analyses. To visualize these interaction results, predicted probabilities were calculated (*Fig*. 1). With increasing age, mortality increased substantially, most prominently after anastomotic leakage.

## Discussion

This population‐based national cohort study suggests that anastomotic leakage results in increased mortality in the prolonged postoperative period. The overall mortality rate was 3·9 per cent in patients with anastomotic leakage. No increased mortality was observed in patients with anastomotic leakage that did not require reintervention. Substantial interaction was detected between leakage and age, indicating that this complication, if present, is more detrimental in older patients.

The major strength of the study is its large, population‐based, cohort design. Estimates of this study are therefore robust, reflecting the whole population of interest, namely patients undergoing anterior resection for rectal cancer today. Small numbers, however, may still have affected some secondary analyses.

The ambiguity in the term anastomotic leakage is problematic for all research aimed at quantifying the rate of leakage^17^. The registry used allows the surgeon to define leakage arbitrarily, thereby threatening the validity of the data on which this study was based. It also provides clinical heterogeneity, perhaps explaining why an association was found only for patients requiring reintervention. In addition, the registry does not allow differentiation between different reinterventions. It would be interesting to demarcate further the group at greatest risk of death after leakage, by separating relaparotomy from simple drainage. Another drawback is the delay in diagnosing leakage; at present, approximately one‐third of all leaks are diagnosed after 30 postoperative days^18^. In addition, as the registry probably underestimates the true incidence even within that time period^19^ ^20^, the estimates presented here are most likely conservative measures.

In the present study, the mortality rates observed following anastomotic leakage are similar to those of other recently published studies on population‐based data^17^ ^21^. The association between anastomotic leak and increased postoperative mortality is reasonable pathophysiologically, and has been shown previously for colorectal surgery^2^ ^22^. For rectal cancer surgery specifically, Snijders and colleagues^23^ tried to quantify this effect in a meta‐analysis of 22 studies; they concluded that one‐third of all postoperative mortality is caused directly by anastomotic leakage. In contrast, the association between age and complication rates, morbidity and mortality is less obvious and difficult to study, partly as randomization of the exposure variable is impossible. Age has, however, been correlated with increased postoperative mortality in studies of colorectal surgery^24^ ^25^.

The driving force behind this increased mortality is not clear, and studies of the correlation between age and complication rates must be well designed to control for the increased co‐morbidity and frailty of the elderly population^26^. However, Marusch and co‐workers^27^ conducted a multicentre study of 309 hospitals, including 19 080 patients who had surgery for colorectal carcinoma, and demonstrated increased complication rates among octogenarians, when controlling for confounders. Aquina *et al*.^28^ evaluated 24 426 patients with stage I–III colonic cancer, while controlling for confounders, and similarly found a dose–response relationship between increasing complication rates and increasing age.

Regarding the association between advanced age and the development of anastomotic leak, the picture is less clear. Den Dulk and colleagues^2^ summarized data from five European trials of colorectal surgery between 1987 and 2003, and found an association between age and mortality after leakage, but not between age and leakage itself. For anterior resection specifically, an observational single‐centre study by Lin *et al*.^29^, of 999 patients in 1993–2003, showed age above the median to be a risk factor for anastomotic leakage in multivariable analysis (OR 2·2, 95 per cent c.i. 1·21 to 3·88). In contrast, Parthasarathy and co‐workers^30^ used prospectively collected data on 17 518 patients in 2013 and found a higher risk of anastomotic leakage among younger patients undergoing colorectal resection. A meta‐analysis by Pommergaard and colleagues^31^ on resection of colorectal cancer found 21 studies that evaluated the effect of age, of which only two showed an association; seven of these studies were used to calculate a pooled OR of 0·99 (0·89 to 1·10).

Although advanced age is not an established risk factor for anastomotic leak, the consequences of leakage seem to be aggravated in the elderly. It is therefore important to consider the use of possible protective measures, including a temporary or definitive stoma. Better individual risk‐profiling is warranted, but the findings of the present study may help to counsel patients and improve shared decision‐making in the preoperative setting.

## Supporting information


**Fig. S1.** Directed acyclic graph (DAG) showing the authors' biological–mechanistic understanding of how possible confounders could influence exposure and outcome. The graph depicts ancestors of outcome (blue), ancestors of both exposure and outcome (red), unobserved ancestors (light grey) and other variables (dark grey), as well as the confounders that need to be addressed in the statistical model (white). In this model, adjustment was necessary only for hospital volume, intraoperative bleeding, ASA score, age and the presence of a diverting stoma in order to yield an unbiased measure of the total effect of anastomotic leakage on postoperative mortality. TME, total mesorectal excision; PME, partial mesorectal excision
