# Supplementary material for: Population‐based cohort study of the impact on postoperative mortality of anastomotic leakage after anterior resection for rectal cancer
Source: BJS Open. 2018 Oct 15;3(1):106–11. doi: 10.1002/bjs5.50106 (PMC6354192; doi:10.1002/bjs5.50106)
Supplement: Supplementary file 1 — Fig. S1. Directed acyclic graph (DAG) showing the authors' biological–mechanistic understanding of how possible confounders could influence exposure and outcome. The graph depicts ancestors of outcome (blue), ancestors of both exposure and outcome (red), unobserved ancestors (light grey) and other variables (dark grey), as well as the confounders that need to be addressed in the statistical model (white). In this model, adjustment was necessary only for hospital volume, intraoperative bleeding, ASA score, age and the presence of a diverting stoma in order to yield an unbiased measure of the total effect of anastomotic leakage on postoperative mortality. TME, total mesorectal excision; PME, partial mesorectal excision [file BJS5-3-106-s001.docx]

BJS5_50106

**Population-based cohort study of the impact on postoperative mortality of anastomotic leakage after anterior resection for rectal cancer**

**P. Boström, M. Haapamäki, J. Rutegård, P. Matthiessen and M. Rutegård**


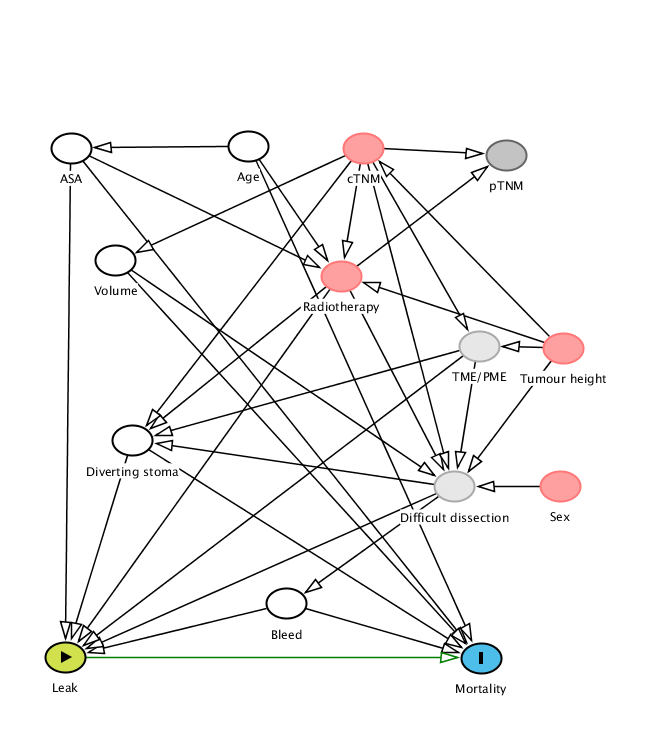


**Fig. S1** Directed acyclic graph (DAG) showing the authors’ biological–mechanistic understanding of how possible confounders could influence exposure and outcome. The graph depicts ancestors of outcome (blue), ancestors of both exposure and outcome (red), unobserved ancestors (light grey) and other variables (dark grey), as well as the confounders that need to be addressed in the statistical model (white). In this model, adjustment was necessary only for hospital volume, intraoperative bleeding, ASA score, age and the presence of a diverting stoma in order to yield an unbiased measure of the total effect of anastomotic leakage on postoperative mortality. TME, total mesorectal excision; PME, partial mesorectal excision
